# A rare case of gradual enlargement of a multifocal myelolipoma of the posterior mediastinum for 12 years after surgical resection of an adrenal myelolipoma

**DOI:** 10.1016/j.ijscr.2018.09.027

**Published:** 2018-09-23

**Authors:** Akira Haro, Takatoshi Fujishita, Haruka Nishikawa, Yoshihiro Taguchi, Takuyuki Kouda, Koutaro Kajiwara, Hideki Makino, Takanori Kanematsu, Yumi Oshiro, Hideki Yokoyama

**Affiliations:** a Department of Thoracic Surgery, Matsuyama Red Cross Hospital, Ehime, Japan; b Department of Respiratory Medicine, Matsuyama Red Cross Hospital, Ehime, Japan; c Department of Diagnostic Pathology, Matsuyama Red Cross Hospital, Ehime, Japan

**Keywords:** Myelolipoma, Posterior mediastinum, Adrenal gland, Surgical resection, Video-assisted thoracic surgery

## Abstract

**Introduction:**

A myelolipoma is a rare benign tumor that is composed of adipose tissue and hematopoietic elements. Myelolipomas most commonly occur in the unilateral adrenal gland. Posterior mediastinal myelolipomas are extremely rare. We herein present a rare case of a multifocal myelolipoma of the mediastinum that gradually enlarged over a 12-year period after surgical resection of an adrenal myelolipoma. This is the first report of multifocal myelolipomas of the posterior mediastinum and adrenal gland.

**Presentation of case:**

A posterior mediastinal tumor was incidentally found by chest X-ray and computed tomography (CT) examination of a 74-year-old woman. The patient had a medical history of resection of a myelolipoma of the left adrenal gland 12 years earlier. We performed tumor extirpation under video-assisted thoracic surgery (VATS). The size of the tumor was 4.5 cm, and the postoperative diagnosis was a myelolipoma.

**Discussion:**

Posterior mediastinal myelolipomas are extremely rare, and only 39 cases of mediastinal myelolipoma have been reported to date. No reports have described a multifocal myelipoma of mediastinal myelolipoma. To our knowledge, this is the first report of multifocal myelipomas of the adrenal gland and posterior mediastinum.

**Conclusion:**

A differential diagnosis of myelolipoma of the posterior mediastinum is important in patients with a history of myelolipoma of the adrenal gland.

## Highlights

• A myelolipoma is a rare benign tumor comprising adipose tissue and normal hematopoietic cells.
• A myelolipoma commonly occurs in the unilateral adrenal gland.
• This is the first rare case of multifocal myelolipomas of the mediastinum and adrenal gland.

## 1 Introduction

A myelolipoma is a rare benign tumor that is composed of adipose tissue and hematopoietic elements and occurs mainly in adrenal gland. Posterior mediastinal myelolipomas are extremely rare, but it should be considered in differential diagnosis of posterior mediastinal tumor. We report the first case of multifocal myelolipomas of posterior mediastinum and adrenal gland. The research work has reported in line with the SCARE criteria [1].

## 2 Presentation of case

**Fig. 1:**
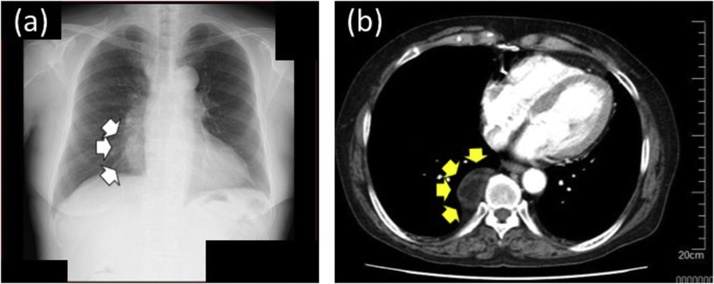
(A) Chest X-ray reveals an abnormal shadow in the right lower lung field (white arrows). (B) Chest CT reveals a 4.5-cm round tumor enhanced by contrast medium in the posterior mediastinal (yellow arrows).

**Fig. 2:**
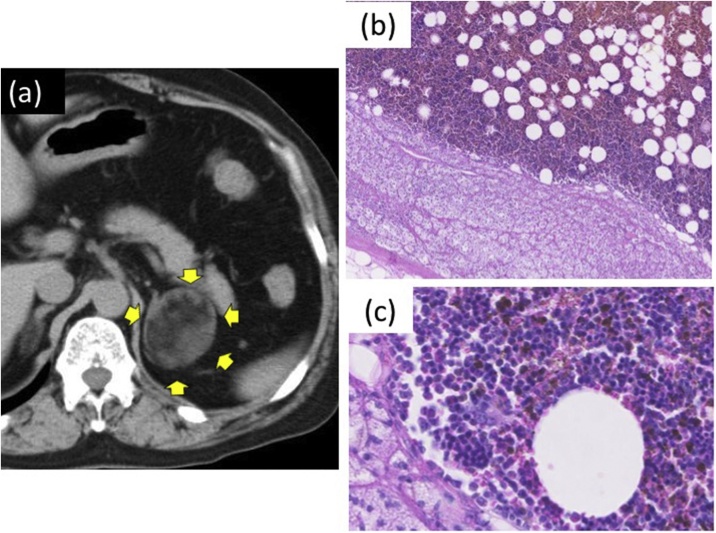
(A) Abdominal CT shows the left surgically resected adrenal myelolipoma (yellow arrows) 12 years previously. (B) Microscopic examination of the left adrenal myelolipoma shows a predominance of adipose tissue and hematopoietic cells. (C) Microscopic examination shows that the hematopoietic cells are mainly lymphocytes.

A posterior mediastinal tumor was incidentally found by a preoperative chest X-ray and CT examination of a 74-year-old woman (Fig. 1A and B) for surgical treatment for calcinosis cutis of the right heel. She was admitted to our hospital without symptoms. She had no relevant family history. However, she had a medical history of surgical resection of a 48 × 40-mm myelolipoma of the left adrenal gland 12 years earlier (Fig. 2).

**Fig. 3:**
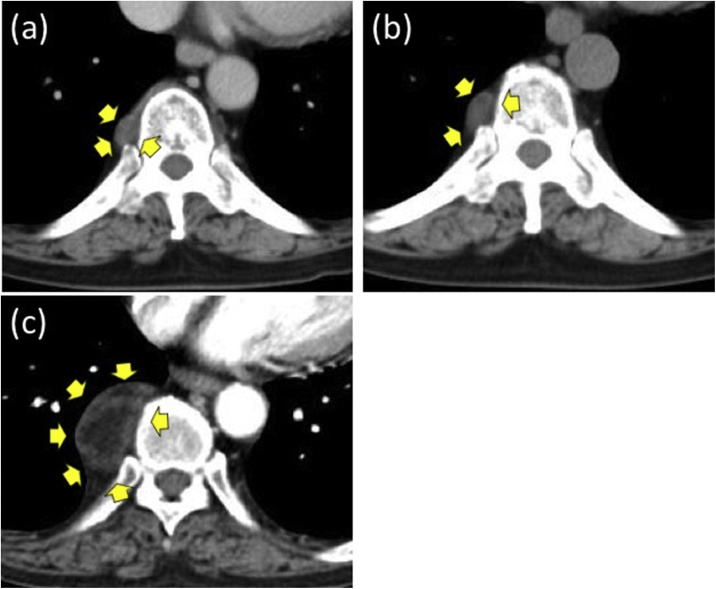
(A) Chest CT shows a posterior mediastinal tumor (yellow arrows) at the time of resection of the left adrenal myelolipoma 12 years previously. (B) The tumor on chest CT examination 10 yeas previously (yellow arrows). (C) The tumor was larger and partially enhanced by contrast medium (yellow arrows) at the first visit to our department.

**Fig. 4:**
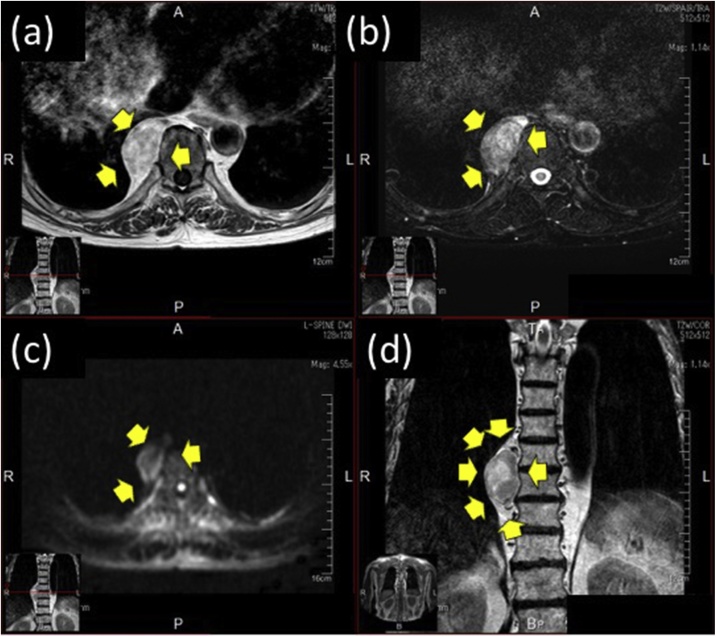
Magnetic resonance images of the posterior mediastinal tumor. (A) The T1 weighted image. (B) The T2 weighted image. (C) Diffusion-weighed image. (D) Magnetic resonance image (coronal plane) reveals the mass attached to the T9 thoracic vertebra.

Upon admission, physical examination and blood examination revealed no abnormal findings. A chest X-ray revealed an approximately 4-cm round tumor in the right lower lung field (Fig. 1A). The round tumor was located in the right posterior mediastinum and showed slight enhancement by contrast medium on chest CT examination (Fig. 1B). It was attached to the T9 thoracic vertebrae, but there were no findings of invasion to surrounding tissues such as the other vertebra or ribs. Retrospective examination of the patient’s previous chest CT scan showed the small tumor at the time of resection of the left adrenal myelolipoma 12 years earlier, and the tumor had since gradually increased (Fig. 3). Magnetic resonance imaging of the tumor revealed high signal on T1-weighted, T2-weighted, and diffusion-weighted images (Fig. 4). The differential diagnoses were lipoma, liposarcoma, angiomyolipoma, neurogenic tumor, myelolipoma, and extramedullary hematopoiesis.

**Fig. 5:**
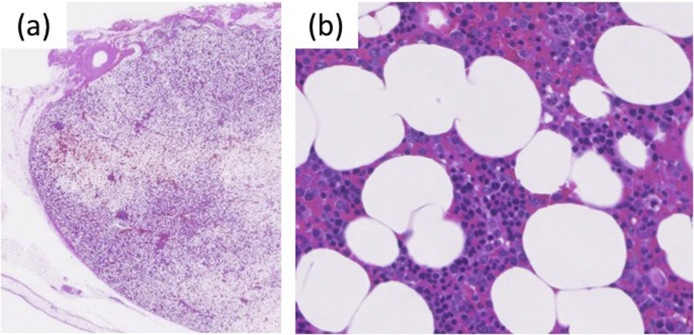
(A) Microscopic examination of the mediastinal myelolipoma covered with parietal pleura. (B) Microscopic examination shows that the mediastinal myelolipoma is predominantly composed of mature adipose tissue and hematopoietic cells.

For diagnosis and treatment, we performed tumor extirpation under VATS. The tumor was covered with parietal pleura and exhibited no invasion to the surrounding tissues. The size of the tumor was 4.5 cm. The operative time was 2 h 47 min, and blood loss was minimal. The postoperative pathological findings revealed mature adipose tissue containing hematopoietic elements (Fig. 5), and postoperative diagnosis was a mediastinal myelolipoma. The patient had an uneventful recovery and was discharged on postoperative day 7. She was still disease free at 6-month follow-up.

## 3 Discussion

A myelolipoma is a rare benign tumor comprising adipose tissue and normal hematopoietic cells. Myelolipomas commonly occur in the unilateral adrenal gland; they rarely develop at extra-adrenal site, with an incidence of 0.08 to 0.2% [2]. Extra-adrenal sites include the presacral resion, retroperioneum, liver, spleen, stomach, lungs, and mediastinum [3]. Only 39 cases of mediastinal myelolipoma have been reported to date [4]. Additionally, no reports have described a multifocal mediastinal myelolipoma. To our knowledge, this is the first report of multifocal myelipomas of the adrenal gland and posterior mediastinum.

Most of mediastinal myelolipoma are detected asymptomatically and incidentally by chest X-ray or CT examination [5]. However, 75% of them were detected symptomatically in one previous study [3]. The patients’ chief complaints in that study were cough, chest pain and dyspnea. The patients comprised 12 women and 16 men with a mean age of 64 years, and most of the mediastinal myelolipoma arose from the posterior mediastinum [3]. Some reports have described bilateral paraventebral myelolipoma [6,7]. In the above-mentioned study, the mass diameter ranged from 1.5 to 25.0 cm with mean diameter of 5.9 cm [3].

CT and magnetic resonance imaging are useful for diagnosis, but a definitive histological diagnosis is difficult to obtain before surgery. Mediastinal myelolipomas are often misdiagnosed as malignant tumors, neurogenic tumors, malignant lymphomas, lipomas, or liposarcomas. CT-guided neddle biopsy is associated with a risk of bleeding and tumor rupture [8]. Minimally invasive VATS is reportedly a better means of both diagnosis and treatment [5].

No standard treatment for mediastinal myelolipoma has been established. Surgical resection is widely accepted as the best treatment for mediastinal myelolipoma [3]. The main surgical indication is considered to be an increase in tumor size or the development of local symptoms [5]. In the present case, the tumor gradually increased in size for 12 years. The retrospective observation period of 12 years in this case is longer than that of 9 years in previous reports [9,10]. In recent years, most of these tumors have been surgically resected by minimally invasive VATS. For early-stage disease, surgical resection with minimally invasive VATS is recommended, based on the assumption that delayed surgery might be associated with greater surgical risks and higher invasiveness [9]. The authors of a previous study proposed that most posterior mediastinal myelolipoma of <8 cm can be successfully resected via VATS [11].

## 4 Conclusion

A differential diagnosis of myelolipoma of the posterior mediastinum is important in patients with a history of myelolipoma of the adrenal gland.

## Conflict of interest

All authors declare no conflicts of interest associated with this manuscript.

## Sources of funding

None.

## Ethical approval

Ethical approval has been exempted by our institution.

## Consent

Written informed consent was obtained from the patient for publication of this case report and accompanying images. A copy of the written consent is available for review by the Editor-in-Chief of this journal on request.

## Author contributions

AH acquired the data and wrote the article. TF, HN, YT, TK, KK, HM, TK, YO and HY coordinated and critically revised the study. All read and approved the final manuscript.

## Registration of research studies

The name of my UIN is researchregistry4037.

## Guarantor

Akira Haro.

## Provenance and peer review

Not commissioned, externally peer-reviewed.

## References

[1] Agha R.A., Fowler A.J., Saeta A. (2016). The SCARE statement: consensus-based surgical case report guidelines. Int. J. Surg..

[2] Fonte J.M., Varma J.D., Kuligowska E. (1999). Thoracic case of the day. Kartagener’s syndrome. AJR Am. J. Roentgenol..

[3] Xiong Y., Wang Y., Lin Y. (2014). Primary myelolipoma in posterior mediastinum. J. Thorac. Dis..

[4] Shi Q., Pan S., Bao Y. (2017). Primary mediastinal myelolipoma: a case report and literature review. J. Thorac. Dis..

[5] Ema T., Kawano R. (2014). Myelolipoma of the posterior mediastinum: report of a case. Gen. Thorac. Cardiovasc. Surg..

[6] Kawanami S., Watanabe H., Aoki T. (2000). Mediastinal myelolipoma: CT and MRI appearances. Eur. Radiol..

[7] Franiel T., Fleischer B., Raab B.W. (2004). Bilateral thoracic extraadrenal myelolipoma. Eur. J. Cardiothorac. Surg..

[8] Rossi M., Ravizza D., Fiori G. (2007). Thoracic myelolipoma diagnosed by endoscopic ultrasonography and fine-needle aspiration cytology. Endoscopy.

[9] Hosaka T., Hata Y., Makino T. (2016). Mediastinal myelolipoma showing gradual enlargement over 9 years: a case report. J. Cardiothorac. Surg..

[10] Himuro N., Minakata T., Oshima Y. (2016). Video-assisted thoracic surgery for primary myelolipoma of the posterior mediastinum. J. Cardiothorac. Surg..

[11] Lin F., Pu Q., Ma L. (2015). Surgical treatment of primary mediastinal myelolipoma. Interact. Cardiovasc. Thorac. Surg..

